# Vertically Aligned CsPbBr_3_ Nanowire Arrays with Template-Induced Crystal Phase Transition and Stability

**DOI:** 10.1021/acs.jpcc.0c11217

**Published:** 2021-02-11

**Authors:** Zhaojun Zhang, Klara Suchan, Jun Li, Crispin Hetherington, Alexander Kiligaridis, Eva Unger, Ivan G. Scheblykin, Jesper Wallentin

**Affiliations:** † Synchrotron Radiation Research and NanoLund, Department of Physics, Lund University, Box 124, Lund 22100, Sweden; ‡ Chemical Physics and NanoLund, Department of Chemistry, Lund University, Box 124, Lund 22100, Sweden; § Centre for Analysis and Synthesis and NanoLund, Department of Chemistry, Lund University, Box 124, Lund 22100, Sweden

## Abstract

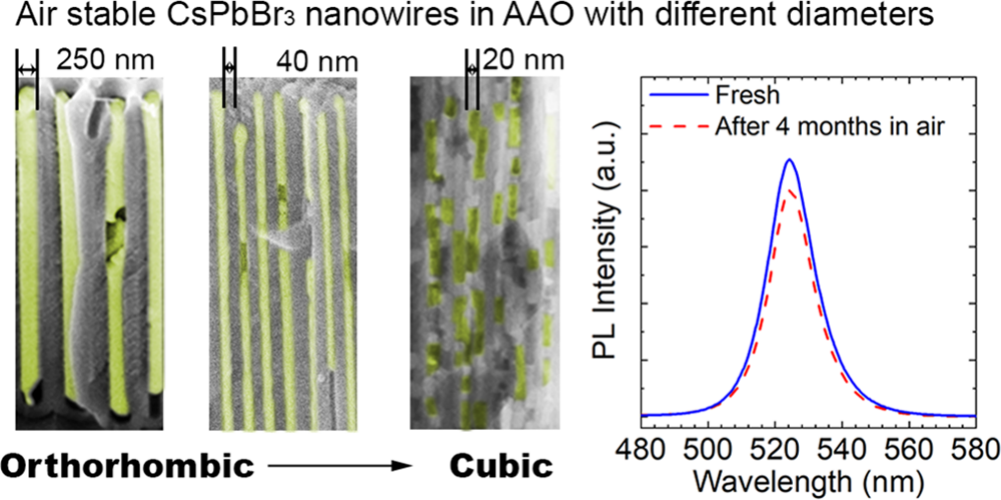

Metal halide perovskites show great promise for a wide range of optoelectronic applications but are plagued by instability when exposed to air and light. This work presents low-temperature solution growth of vertically aligned CsPbBr_3_ nanowire arrays in AAO (anodized aluminum oxide) templates with excellent stability, with samples exposed to air for 4 months still exhibiting comparable photoluminescence and UV stability to fresh samples. The single-crystal nanowire length is adjusted from ∼100 nm to 5 μm by adjusting the precursor solution amount and concentration, and we observe length-to-diameter ratios as high as 100. Structural characterization results indicate that large-diameter CsPbBr_3_ nanowires have an orthorhombic structure, while the 10 nm- and 20 nm-diameter nanowires adopt a cubic structure. Photoluminescence shows a gradual blue-shift in emission with decreasing nanowire diameter and marginal changes under varying illumination power intensity. The CsPbBr_3_-nanowires/AAO composite exhibits excellent resistance to X-ray radiation and long-term air storage, which makes it promising for future optoelectronic applications such as X-ray scintillators. These results show how physical confinement in AAO can be used to realize CsPbBr_3_ nanowire arrays and control their morphology and crystal structure.

## Introduction

During the past several years, there has been a surge in the development of metal halide perovskites (MHPs), particularly for optoelectronic applications such as photovoltaics, luminescence, and photon detection, due to their excellent properties including strong optical absorption, long carrier diffusion lengths, high photoluminescence quantum yields, tunable band gaps, and solution processability.^1−3^ The main limitation hindering widespread commercial deployment of MHP devices is instability when exposed to moisture, heat, and light.^4,5^ Generally, inorganic MHPs show a much better stability than organic hybrid MHPs, and within this group of materials, cesium lead bromide CsPbBr_3_ has achieved excellent performance in the fields of photovoltaics,^6^ light emitting diodes (LEDs),^7,8^ photodetectors,^9^ radiation detectors,^10,11^ and lasers.^12^ Still, stability remains a challenge for all MHPs.^4,5^

The most common approach for improving MHP stability is through various chemical modifications,^13^ but promising results have also been reported using physically confined nanowires (NWs).^14^ Semiconductor NWs additionally allow new possibilities unavailable for bulk crystals, as demonstrated in established group IV and III–V semiconductors. Studies have shown that the basic material properties of NWs, for instance, the crystal phase and the thermal conductivity, can be fundamentally different from the corresponding bulk material.^15,16^ In particular, the light absorption and emission in arrays of vertically aligned NWs have shown impressive performance in solar cells^17^ and LEDs.^18^ Thus, growing MHPs in the form of physically confined vertically aligned NW arrays could both give improved stability and allow new design possibilities and functionalities for devices.

The growth of CsPbBr_3_ NWs and their various optoelectronic applications have been explored, e.g., for lasing,^12^ multicolor displays,^19^ control of emission anisotropy,^20^ and light guiding^21^ as well as photodetection.^22−24^ However, most of these studies used unprotected in-plane NWs with horizontal alignment.^12,19−28^ A few studies have investigated vertically aligned MHP NW arrays, which have been proven to have high light extraction capability for LED devices^14,29^ and high resolution for pixel image sensors.^30^ So far, studies on vertically aligned CsPbBr_3_ NW arrays are limited. To our knowledge, only Meng *et al.* have used the vapor–liquid–solid mechanism and high-temperature vapor growth to achieve vertical CsPbBr_3_ nanowires.^22^

Here, we report a low-temperature solution growth of vertically aligned CsPbBr_3_ NWs arrays with excellent stability using anodized aluminum oxide (AAO) templates. AAO is a commonly used template for nanocrystal synthesis, and it has also been successfully applied for growth of MHP nanocrystals.^31−35^ Using this method, the NW arrays will have excellent vertical alignment. The diameter of the NWs can also be easily adjusted. However, the growth behavior and mechanism of micrometer-long CsPbBr_3_ NWs in AAO pores from a solution precursor have not been clearly investigated.

In this work, the growth behavior of CsPbBr_3_ NWs in a micrometer-thick AAO template from solution is clearly elucidated based on the solubility properties of the precursor. The diameter dependence of the structural and photoluminescence (PL) properties of CsPbBr_3_ NWs is also investigated. The CsPbBr_3_ NWs exhibit a cubic-like structure below 20 nm, while larger diameter pores lead to NWs adopting the expected orthorhombic structure (40–250 nm). The novel combination of CsPbBr_3_, one of the most stable in the MHP class, with the physical protection of the AAO gives the CsPbBr_3_ NWs an exceptional stability to ambient air exposure. The CsPbBr_3_-NWs/AAO samples exposed to air for 4 months still exhibit comparable photoluminescence and UV stability to fresh samples. These CsPbBr_3_-NWs/AAO composites provide new possibilities to realize functional vertically structured CsPbBr_3_ NW array-based optoelectronic devices with sufficient stability to be of technological importance.

## Methods

### Growth of CsPbBr_3_ NWs

All the AAO templates were purchased from InRedox Company. The AAO templates with different nominal diameters of 10, 20, 40, 80, 160, and 250 nm all have the same thickness of 5 μm and were made on a 100 μm-thick Al substrate. CsBr (99.9%), PbBr_2_ (98%), and dimethyl sulfoxide (DMSO) (AR, 99.9%) were purchased from Sigma-Aldrich. Different concentrations (0.1, 0.2, 0.3, and 0.45 mol/L) of precursors were made by dissolving 106, 212, 318, and 477 mg of CsBr and 183.5, 367, 550.5, and 825.75 mg of PbBr_2_, respectively, in 5 mL of DMSO by vigorous stirring for 2 h until a clear solution was obtained. The AAO templates were ultrasonically cleaned in ethanol for 1 min and then dried by N_2_. Deposition was done by putting 50 μL of precursor using a pipette on a cleaned 1 × 1 cm^2^ AAO surface, waiting 1 min, and spin-coating with a speed of 3000 rpm for 1 min. For growing longer NWs, an extra precursor was added on the template surface. Finally, the template was put on a hot plate at 70 °C (temperature error ± 0.1 °C) for 30 min.

### Scanning Electron Microscopy (SEM)

Cross-sectional SEM images were observed from the crack produced by bending the sample on the 90° edge of the sample holder. The CsPbBr_3_ NWs were distributed randomly between the two sides, and thus there were several empty pores in the cross-sectional SEM images taken from one side. The instrument used was a Hitachi SU8010 Cold Field Emission SEM.

### Energy Dispersive Spectroscopy (EDS)

EDS was performed using a Zeiss Geminis 500 electron microscope with an acceleration voltage of 10 keV.

### Transmission Electron Microscopy (TEM)

The samples were prepared by using a knife to scrape the AAO membrane with CsPbBr_3_ NWs inside. Then, a TEM Cu grid was put on the AAO surface since some AAO fragments stick on the carbon film of the TEM grid. The instrument used was a 300 kV JEM-3000F in TEM mode and HAADF-STEM mode.

### X-ray Diffraction (XRD)

Measurements were performed using an STOE STADI MP diffractometer with a Cu anode operating at 40 kV and 40 mA as an X-ray source in reflection mode. In this geometry, the scattering vector was parallel with the AAO pores, and the measurements probe crystal planes that are approximately orthogonal to the pore axis (i.e., parallel with the substrate). A DECTRIS MYTHEN 1K detector was set with a scanning step width of 0.2° and a counting time of 30 s per step. The pixel detector gives a precise angular resolution of 0.03° FWHM even with a scanning step of 0.2°.

### Photoluminescence (PL)

For all the measurements, the excitation laser was incident to the top surface of the CsPbBr_3_-NWs/AAO sample. Time-dependent PL intensity was measured using an inverted wide-field fluorescence microscope (Olympus IX-71) under a 485 nm laser and 900 μm^2^ spot size. The excitation power-dependent PL spectra were measured with excitation by a 378 nm laser. The X-ray scintillation spectra were collected with OceanOptics QE65 Pro spectrometer and a microfocus X-ray source (Moxtek, Inc., tungsten, 40 kV, 300 μA).

## Results and Discussion

The obtained NWs are crystallized by solvent evaporation of a precursor solution. The precursor is made by mixing equal molar CsBr and PbBr_2_ in DMSO to obtain pure-phase CsPbBr_3_. There are three compounds in the CsBr-PbBr_2_ system with different stoichiometric ratios (CsPbBr_3_, CsPb_2_Br_5_, and Cs_4_PbBr_6_).^36−39^ In this work, the obtained NWs are pure CsPbBr_3_ without the formation of Cs_4_PbBr_6_ and CsPb_2_Br_5_ as verified by the energy-dispersive spectroscopy (EDS) test in Figure S1 in the Supporting Information. All the processing is done in air.

### Growth of Vertically Aligned CsPbBr_3_ NWs Arrays with Different Lengths

#### Controlling the NW Length by Tuning the Precursor Concentration

**Figure 1:**
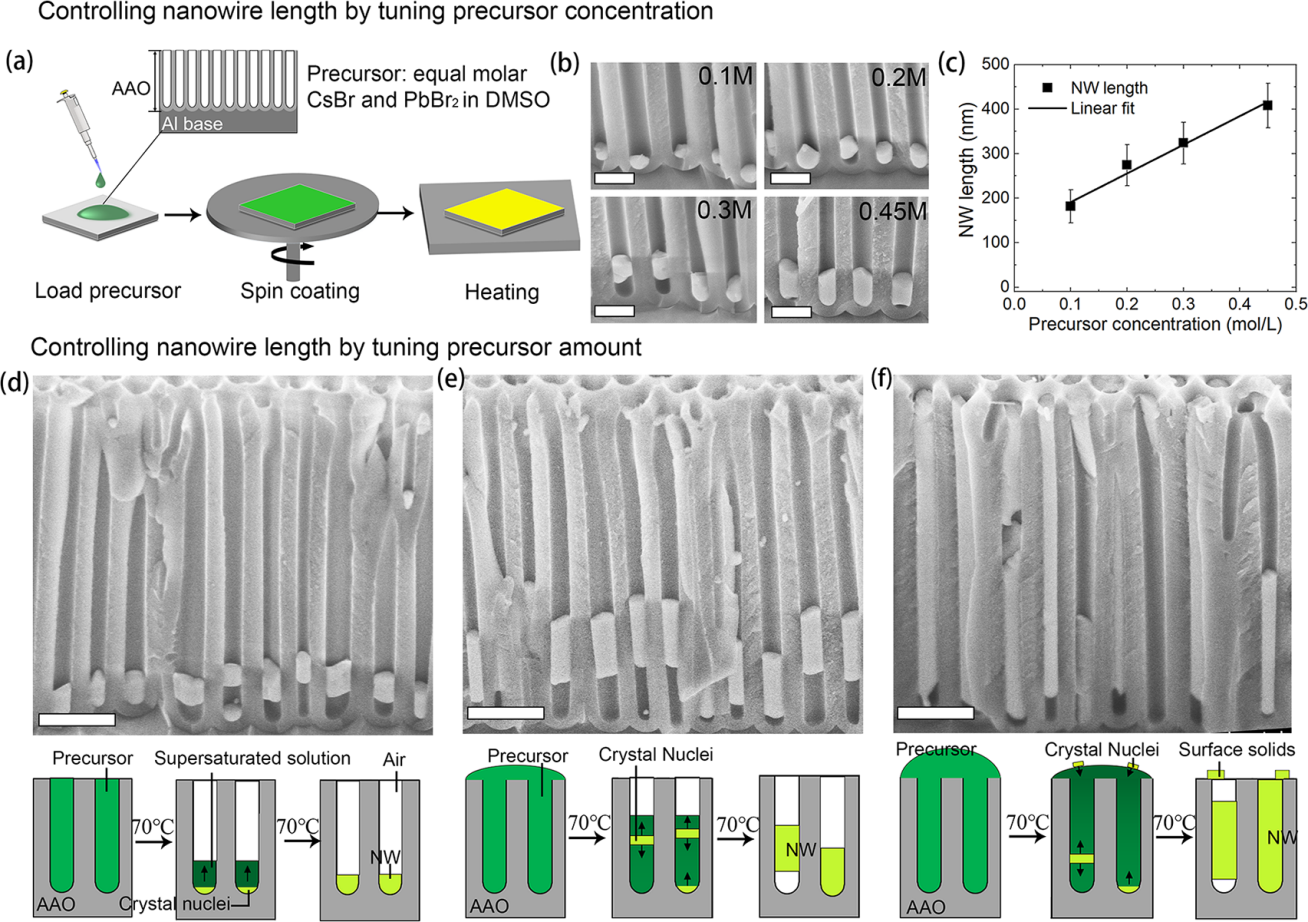
Growth of CsPbBr_3_ NWs using AAO pores with a diameter of 250 nm and length of up to 5 μm. (a) Schematic diagram of growth process. (b) Cross-sectional SEM of NWs grown by using increasing precursor concentration. Scale bars: 500 nm. (c) Measured NW length versus precursor concentration, with a linear fit. (d–f) Cross-sectional SEM and schematic growth process of NWs grown by increasing precursor amount. Scale bars: 1 μm. From (d) to (f): first, spin-coating with 3000 rpm and 1 min to remove the air inside the pores and fill the pores with the precursor. For growing longer NWs, further (e) 5 μL and (f) 10 μL of 0.45 M precursor were added on the template (1 cm^2^) surface.

As shown in Figure 1a, the AAO pores are infiltrated with the precursor by spin-coating. Then, the sample is heated on a hot plate at 70 °C for 30 min and the NWs grow inside the pores. Cross-sectional scanning electron microscopy (SEM) images of CsPbBr_3_ NWs grown by using different concentrations of precursor solutions are shown in Figure 1b. The faceted top of the NWs indicates single-crystal growth, while the other sides of the NWs are shaped after the pores. Thus, the NWs grow from the bottom of AAO pores, showing that the precursor completely fills the pores and displaces the air that was initially there. As shown in Figure 1c, the NW length is proportional to the precursor concentration. Nanocrystals with a size that is even smaller than the pore diameter can also be obtained by further lowering the concentration (Figure S2 in the Supporting Information).

The longest NW length is limited by the maximum precursor concentration with a fixed AAO pore length. The solubility of PbBr_2_ in DMSO is about 2 M at room temperature, but the solubility of pristine CsBr is only about 0.2 M.^40,41^ Although the existence of PbBr_2_ will increase the solubility of CsBr, the solubility limit is still only about 0.45 M when dissolving equal molar fractions of CsBr and PbBr_2_ in DMSO.^40,41^ This value is much smaller than the solubility limits of other MHP materials. For instance, methylammonium lead iodide (MAPbI_3_) has a maximum solubility of ∼2 M in γ-butyrolactone.^42^ For CsPbBr_3_, even when the AAO pore is completely filled with the maximum precursor concentration (∼0.5 M), the NW length is only 6% of the whole pore length (see the detailed explanation in Section S1 in the Supporting Information). Using this method, increasing the pore length could be a way to increase the NW length, but the low filling rate (6%) of the pores may cause some problems for practical device application.

#### Controlling the NW Length by Tuning the Precursor Amount

To increase the filling rate of the AAO pore by CsPbBr_3_, we first repeated the spin-coating several times. However, the precursor solution dissolves the previously formed NWs since the precursor is saturated for CsBr but not saturated for PbBr_2_ according to the above analysis. Instead, we increased the precursor amount. As seen from Figure 1, with increasing precursor amount, the CsPbBr_3_ NWs grow longer from (d) 400 nm to (e) 1 μm and finally to (f) 5 μm, which completely fills the AAO pores. Note that some empty pores and broken NWs are due to the substrate cleaving process used to see the NWs in the SEM.

For all the samples in Figure 1d–f, the first step spin-coating was used to first make sure that the pores are filled with the precursor. In addition, for the samples in Figure 1e,f, extra precursor was added to the AAO surface as a reservoir. Using this strategy, micrometer-long CsPbBr_3_ NWs with diameters of 40, 80, and 160 nm were also successfully grown (Figure S3 in the Supporting Information). The largest aspect ratio of a NW with a diameter of 40 nm is about 100, which is much higher than those of previously reported MHP NWs grown in AAO.^14,32−34^ However, with an even smaller pore diameter, it is more difficult for the precursor to reach the nucleated crystal and make the NWs completely fill the pores.

#### Growth Model Analysis

While the sample is being heated, the solvent gradually evaporates, and eventually a supersaturated solution satisfying the nucleation condition is obtained (Figure 1d–f, bottom row). At this stage, the solution volume has been significantly reduced. The crystal nuclei form where supersaturation is the highest or the nucleation barrier is the lowest. Once a stable crystal nucleus is formed, it can grow without the nucleation barrier until the precursor solution is depleted. As shown in Figure 1d, after spin-coating, the infiltrated precursor has almost the same volume as the pores. As the solvent evaporates, the solution collects at the bottom. The NWs grow from the pore bottom since it has a lot of interface area with a lower nucleation barrier. The resulting CsPbBr_3_ NWs are up to 400 nm long, limited by the amount of infiltrated precursor. When the amount of precursor exceeds the pore volume, as shown in Figure 1e,f, the materials for crystal growth can be supplied continuously from mass transfer within the solution. However, the larger volume of supersaturated solution also means a longer growth time and larger local differences in supersaturation. Thus, there are more possible places for nucleation. Consequently, there is a larger variation of the NW position within the pores, as shown in Figure 1e. Also, there will be more surface growth owing to the excess supersaturated solution, as shown in Figure 1f.

In summary, different from other MHP materials such as MAPbI_3_, which can obtain a high precursor concentration,^34^ the low maximum concentration of precursor for growing pure-phase CsPbBr_3_ from solution makes the continuous supply of precursor to the AAO pores essential for growing micrometer-long NWs inside AAO with a high filling rate of the pores.

### Diameter-Dependent Structural Properties of CsPbBr_3_-NWs/AAO

**Figure 2:**
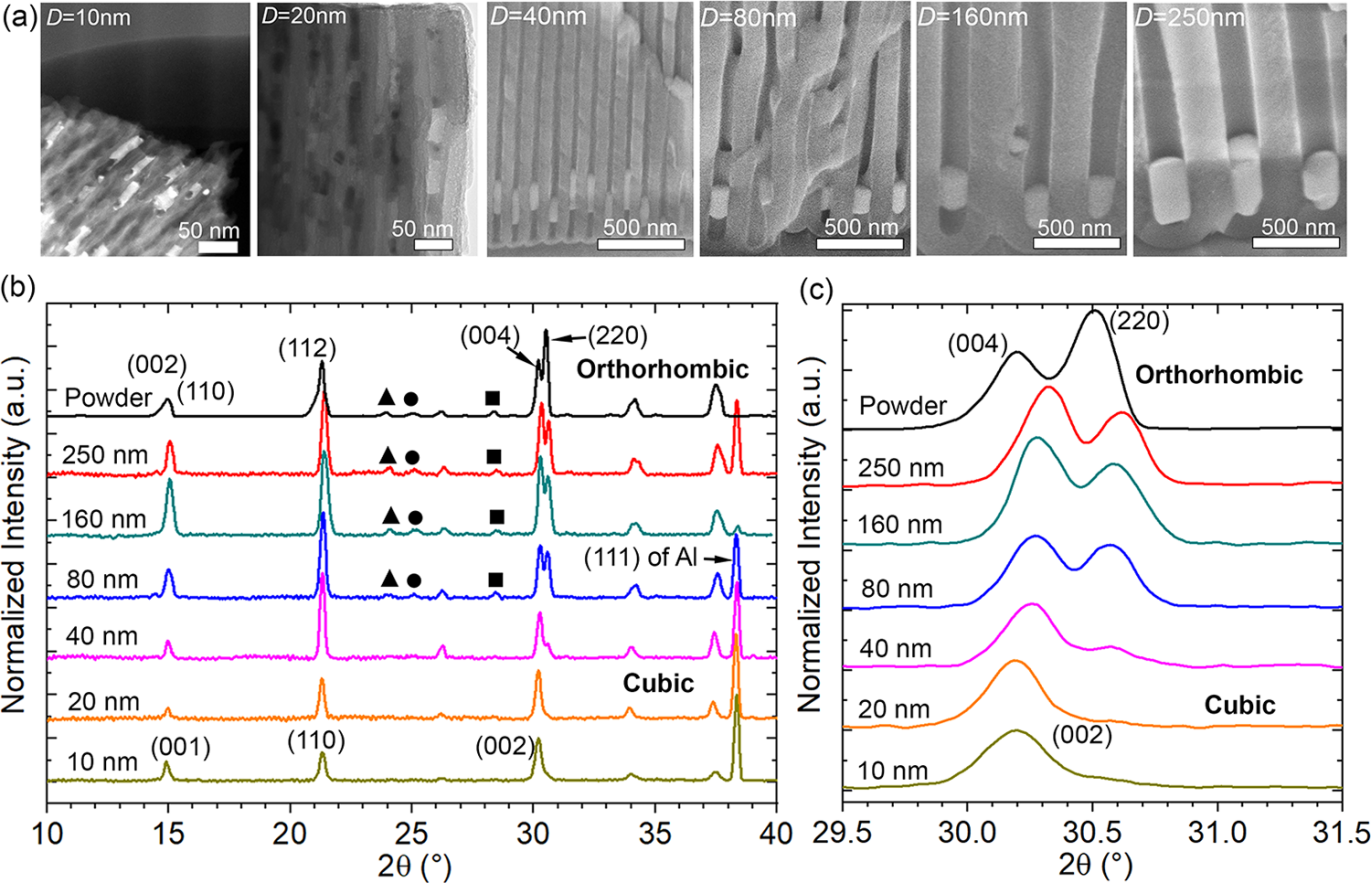
Crystal structure of CsPbBr_3_-NWs/AAO with varying diameters. (a) STEM (10 nm), TEM (20 nm), and SEM (40–250 nm) images of CsPbBr_3_-NWs/AAO with diameter *D* from 10 to 250 nm. (b) XRD patterns of CsPbBr_3_-NWs/AAO with different diameters. (c) Enlarged XRD patterns in the range from 29.5° to 31.5°. The (004) and (220) peaks of orthorhombic-phase CsPbBr_3_ gradually transform to the (002) peak of cubic-phase CsPbBr_3_.

To study the effect of NW diameter on the structural and optical properties, we grew CsPbBr_3_ NWs with diameters ranging from 10 to 250 nm. The photos of the samples are shown in Figure S4a in the Supporting Information. For simplicity, the nominal diameter of the AAO is used to name the as-grown CsPbBr_3_-NWs/AAO samples, although the real diameters of NWs have some variation compared to the nominal values due to the inhomogeneity of AAO pores, as seen from the cross-sectional electron microscopy images of NWs with different diameters shown in Figure 2a. The following measurements are based on CsPbBr_3_-NWs/AAO composites, not on extracted NWs. As shown in Figure S4b in the Supporting Information, there are almost no surface solids, which ensures that the collected X-ray diffraction (XRD) patterns come from the CsPbBr_3_ NWs.

The XRD patterns were measured, and the results are displayed in Figure 2b,c, together with the powder diffraction pattern of a solution-grown bulk CsPbBr_3_ crystal. Bulk CsPbBr_3_ crystallizes in the orthorhombic phase (*Pnmb*) at room temperature, which transforms to tetragonal (*P4mbm*) at 80 °C and cubic (*Pm-3m*) at 130 °C.^11,43^ Reported powder XRD patterns of bulk orthorhombic, tetragonal, and cubic CsPbBr_3_ are also shown in Figure S5a in the Supporting Information.^44^ As shown in Figure 2c and Figure S5a in the Supporting Information, for NWs with diameter *D* from 80 to 250 nm, the measured XRD scans agree well with the patterns of the orthorhombic CsPbBr_3_. The first evidence is that there is a split between the (220) and (004) peaks at around 30.5°. The second evidence is that the diffraction peaks (●) at ∼25° only appear in the orthorhombic phase, not in the tetragonal or cubic phase (Figure S5b in the Supporting Information). The large full width at half-maximum (FWHM) values of these three peaks (marked with ▲, ●, and ■) in the range from 23° to 29° are due to the overlapping of adjacent peaks. Compared with the powder XRD patterns of bulk CsPbBr_3_, the NWs show a stronger (004) peak intensity, which suggests a certain degree of preferential orientation of the crystal axis with respect to the pores.

As shown in Figure 2c, with decreasing diameter from 40 to 10 nm, the relative intensity of the (220) diffraction peak decreased significantly, while the orthorhombic (004) peak gradually transformed to the (002) peak of the cubic phase. Similarly, the three diffraction peaks (marked with ▲, ●, and ■ in Figure 2b) which are characteristic for the orthorhombic-phase CsPbBr_3_, are also absent in the patterns of CsPbBr_3_-NWs/AAO with diameters *D* of 20 and 10 nm. The only shown diffraction peak is at about 30.2°, which is also consistent with the pattern of cubic CsPbBr_3_. Here, we should note that the size-induced broadening is comparatively small, as shown in Figure 2c, which is because the measurements were done with the scattering vector parallel with the NWs, so the size broadening is related to the longer NW length rather than the small diameter. We conclude that the CsPbBr_3_ NWs with a diameter from 40 to 250 nm have the bulk orthorhombic phase, but with further decreasing diameter, when the diameter is below 20 nm (which is close to the size 15 nm of cubic-structure CsPbBr_3_ colloidal nanocrystals^8^), the CsPbBr_3_-NWs/AAO gradually distort to adopt a higher symmetry cubic phase.

To our knowledge, this is the first observation of nanoconfinement-induced evolution of the orthorhombic to cubic structure of CsPbBr_3_ at room temperature. As is known from previous studies, with decreasing size, the relative importance of the surface energy will increase; as a result, cubic-phase CsPbBr_3_ will be stable at room temperature.^7,8,45^ Stabilized cubic-phase CsPbBr_3_ colloidal nanocrystals have been synthesized and stabilized based on this, but it requires organic ligands (such as oleylamine and oleic acid) to prevent aggregation. In our work, the cubic CsPbBr_3_ NWs are stabilized by the physical nanoconfinement of AAO. Additionally, Zeng *et al.*(7) mentioned that room-temperature synthesized colloidal CsPbBr_3_ nanocrystals do not have the cubic structure despite a small size of 10 nm. However, the growth temperature in this work (70 °C) is much lower than the synthesis temperature (140–200 °C) of cubic-phase CsPbBr_3_ colloidal nanocrystals.^8^ It is possible that the lattice distortion and strain of the NWs in AAO contribute to the stabilization of cubic-phase CsPbBr_3_ NWs in our samples based on the previous studies of strain-stabilized cubic CsPbI_3_ and FAPbI_3_ grown from low-temperature solution.^46,47^

**Figure 3:**
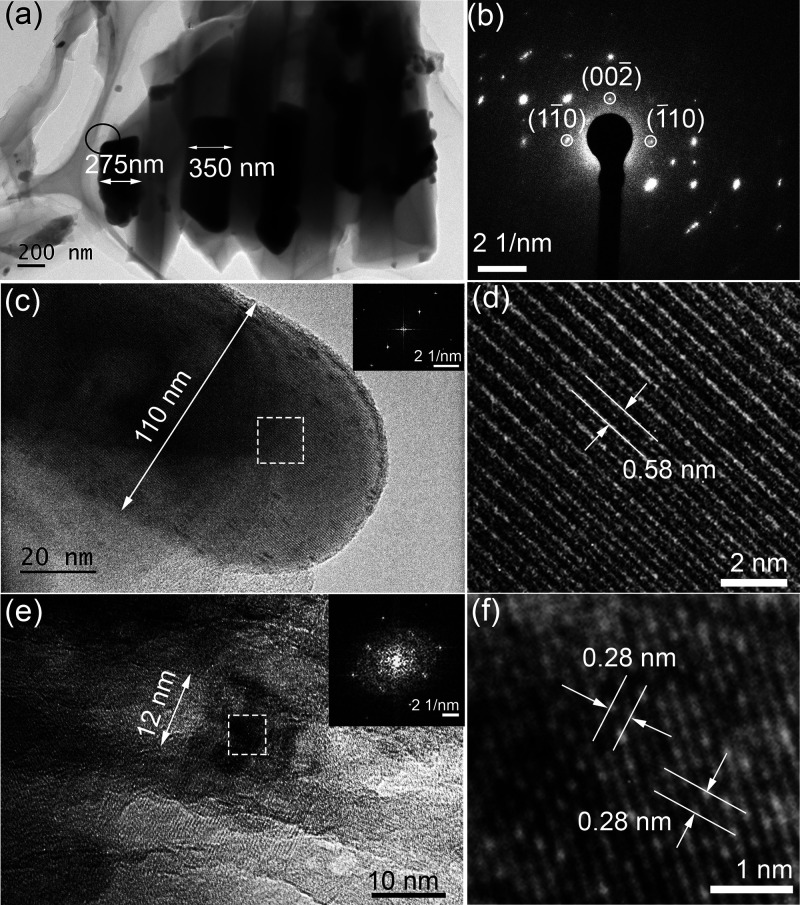
(a) TEM image and (b) SAED pattern of CsPbBr_3_ NWs with diameters of 275 and 350 nm in AAO. (c) HRTEM of the bottom part of a CsPbBr_3_ NW at the edge of a broken piece of AAO. The inset shows the FFT profile of the selected area. (d) Enlarged image of the selected area in (c). (e) HRTEM of a CsPbBr_3_ NW in AAO with a diameter of 12 nm. The inset shows the FFT profile of the selected area. (f) Enlarged image of the selected area in (e).

To examine whether the NWs are single-crystalline, transmission electron microscopy (TEM) was performed. MHPs are easily damaged under exposure to a high intensity electron beam. Since the CsPbBr_3_ NWs could not be extracted from the AAO, they were mostly examined inside this insulating matrix. Compared to the TEM of colloidal CsPbBr_3_ nanocrystals, the AAO increases the charging and makes the nanowires more susceptible to damage by the electron beam. Nevertheless, Figure 3a shows the low-magnification TEM of CsPbBr_3_ NWs in AAO, which clearly shows that the NWs have facets aligned with the pores. The electron diffraction pattern of a selected area of the NWs is shown in Figure 3b, which indicates that the CsPbBr_3_ NW is single-crystalline with growth along the [001] direction and has the orthorhombic phase, in line with the XRD results. A high-resolution TEM image of a single CsPbBr_3_ NW extracted from the AAO is shown in Figure 3c. The hemispherical end of the NW shows that it is grown from the pore bottom. The inset FFT indicates that this NW is also single-crystalline, and the pattern in the FFT is consistent with the orthorhombic structure. As Figure 3d shows, the lattice spacing is about 0.58 nm, which agrees with the lattice spacing of the (110) planes of orthorhombic CsPbBr_3_. This means that one of the sidewall planes of the NWs is (110). The 10 nm NWs are shown in Figure 3e,f and Figure S6 in the Supporting Information. The lattice spacing is measured to be about 0.28 nm (Figure 3f), close to the (002) plane of cubic CsPbBr_3._ Thus, the TEM results show that the CsPbBr_3_ NWs are single-crystalline with crystal structures that agree with the XRD measurements.

### Diameter-Dependent Photoluminescence of CsPbBr_3_-NWs/AAO

**Figure 4:**
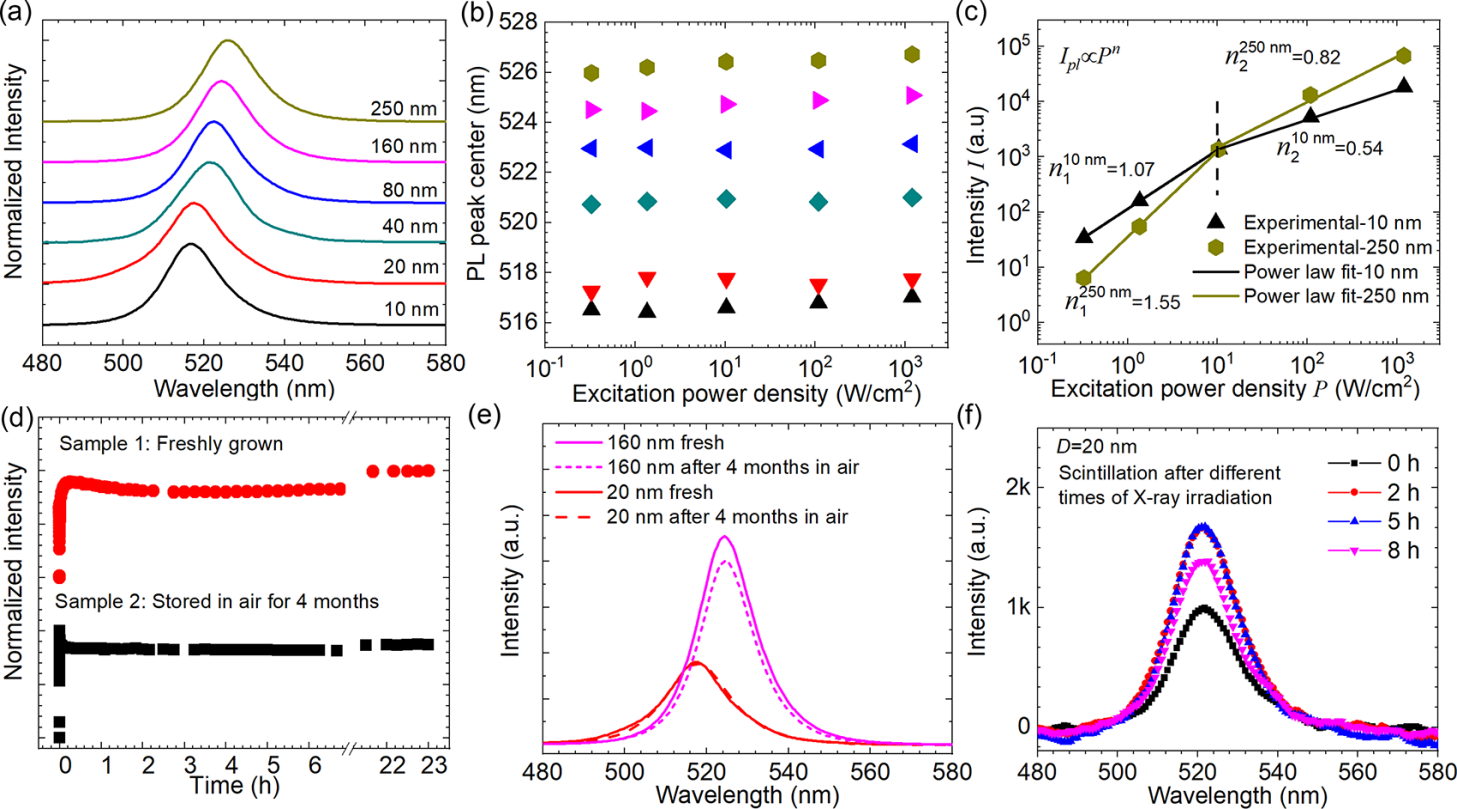
Diameter-dependent photoluminescence properties of CsPbBr_3_-NWs/AAO. (a) Normalized and vertically offset PL spectra (excitation: *P* = 110 W/cm^2^, 378 nm laser). (b) Excitation power-dependent emission center positions (semilog scale). (c) Excitation power-dependent PL intensity of 10 and 250 nm samples (log–log scale), with power law fits. (d) Time-dependent PL intensity test under 1 sun (0.1 W/cm^2^) illumination by a 485 nm laser for a fresh sample and another sample stored in air after 4 months (both 80 nm in diameter). The two curves were vertically offset for clarity. (e) Comparison of PL spectra of 20 nm- and 160 nm-diameter CsPbBr_3_-NWs/AAO before and after 4 months storage in air (excitation: 110 W/cm^2^, 378 nm laser). (f) X-ray scintillation spectra of a 20 nm sample after irradiation of 29 mGy/s X-rays for 0, 2, 5, and 8 h.

The optical properties are crucial for most device applications of MHPs. The PL spectra of CsPbBr_3_-NWs/AAO with diameters from 10 to 250 nm under excitation by a 378 nm laser, with power densities varying from 10^–1^ to 10^3^ W/cm^2^, are displayed in Figure S7 in the Supporting Information. Figure 4a shows the normalized photoluminescence (PL) spectra obtained under an excitation power density *P* of 110 W/cm^2^.

As shown in Figure 4a, with decreasing diameter, the PL of CsPbBr_3_-NWs/AAO has a gradual blue-shift from 526 to 517 nm. To understand this result, quantum confinement was first excluded since the NW diameters are much larger than the Bohr-exciton radius for CsPbBr_3_ (∼7.5 nm).^48^ One possible reason for the blue-shift is, with decreasing diameter, the larger surface-to-volume ratio will create more surface of CsPbBr_3_ NWs. Since CsPbBr_3_ is a relatively soft material,^49,50^ more surface between AAO and NWs means more lattice distortion, which affects the energy band structure of CsPbBr_3_ and possibly causes the emission wavelength blue-shift.^39,51^ A second possible reason is strain between the NWs and the AAO pores, like the explanations of PL blue-shift in CsPbBr_3_ NWs in previous reports.^20,21,26,48,50^

The excitation power-dependent PL emission peak positions of the NWs with different diameters are shown in Figure 4b. For all NWs, the PL peak positions and widths show almost no change with increasing excitation power (Figure S8 in the Supporting Information), which indicates a high crystal quality of CsPbBr_3_ NWs. The power dependence of PL intensity is related to trap states and the surface (interface) nonradiative recombination process in CsPbBr_3_-NWs/AAO composites.^52^ The relation of the integrated PL peak, *I*_pl_, versus the excitation power density, *P*, of 10 and 250 nm CsPbBr_3_-NWs/AAO is shown in Figure 4c (the results of other diameter samples are shown in Figure S9 in the Supporting Information). With excitation power density increasing from 0.3 to 10 W/cm^2^, a power law fit showed an exponent 1 < *n*_1_ < 2 (*n*_1_^10nm^ = 1.07, *n*_1_^250nm^ = 1.55), which indicates that the emission is dominated by exciton recombination.^53^ From 10 to 1200 W/cm^2^, *n*_2_ < 1 (*n*_2_^10nm^ = 0.54, *n*_2_^250nm^ = 0.82), which suggests stronger nonradiative recombination via trap states under higher excitation power density.^53^

### Stability of CsPbBr_3_-NWs/AAO to Air and X-ray Exposure

The main limitation of MHP devices is their instability when exposed to moisture, heat, and light.^4,5^ CsPbBr_3_ nanocrystals, even though they are one of the most stable MHP materials, are normally reported to be sensitive to light irradiation and moisture in the environment.^54−56^ Thus, an important question is if the AAO matrix physically protects the CsPbBr_3_ nanowires. First, we took two 80 nm samples, one fresh and one stored in air for 4 months and compared the UV stability of these samples by measuring the time-dependent PL intensity for 23 h, under constant 1 sun (0.1 W/cm^2^) excitation by a 485 nm laser, as shown in Figure 4d. Both samples show a fast increase from UV excitation during the first 1 s (Figure S10a in the Supporting Information), after which the PL intensity becomes stable after several minutes (Figure S10b in the Supporting Information). After irradiation for 23 h, both samples display a slightly increased intensity, possibly due to defect curing.^57,58^

Next, we measured one 20 nm-diameter sample and one 160 nm-diameter sample before and after 4 months of storage, the length of our project so far, in an air environment (about 45% relative humidity). Surprisingly, we could not observe a significant decrease in the PL (Figure 4e) intensity.

We also repeated the XRD measurements, and we could not find any extra diffraction peaks corresponding to degradation products (Figure S11). The structure of the CsPbBr_3_ NWs, cubic-like for 20 nm and orthorhombic for 160 nm, also remained the same as when they were initially grown. Ideally, the effect of the physical protection of the NWs should be investigated by comparing with isolated NWs but extracting the NWs in a nondestructive manner is very challenging. Compared to, for instance, CsPbBr_3_ films that displayed 50% PL degradation from 5 h of blue LED irradiation,^54^ the CsPbBr_3_-NW/AAO shows much higher photostability against UV irradiation owing to the encapsulation effect of AAO.

The air-stable CsPbBr_3_-NWs/AAO composite has promising applications in future vertical optoelectronic devices. One possibility is X-ray scintillation since CsPbBr_3_-QDs-composed films have shown excellent X-ray scintillation performance.^59^ For this application, resistivity to X-ray radiation damage is always important. Therefore, we measured the X-ray scintillation of the 20 nm-diameter samples under irradiation of X-rays (29 mGy/s) for 8 h, as shown in Figure 4f. After irradiation for 8 h, the scintillation intensity did not show a decrease, but rather a slight increase, which is possibly due to defect curing.^57,58^ These results indicate promising applications of the CsPbBr_3_ NWs-AAO samples for X-ray scintillation, which will be the focus of our future studies.

## Conclusions

In conclusion, we have shown how AAO can be used as a template to successfully grow micrometer-long vertically aligned CsPbBr_3_ NW arrays from solution. The low solubility of their precursors makes it more challenging to fill the nanopores with CsPbBr_3_ compared with other MHP materials, but it can be overcome by supplying sufficient solution. The diameter and the length can be independently controlled, which is a crucial requirement for devices. With decreasing diameter, the CsPbBr_3_ NWs show a gradual PL blue-shift and a gradual change in the crystal structure from orthorhombic to cubic. The physical confinement of the CsPbBr_3_ NWs in the AAO gives a remarkable stability to long-term air storage. Any significant degradation of these structures is not observed within the present length of the project, 4 months, despite storing the sample in ambient air. Additionally, the CsPbBr_3_-NWs/AAO composite shows good resistance to X-ray exposure, which makes it promising for applications in X-ray scintillation. The physical protection could be further enhanced by capping the open end of the AAO, and the method is fully compatible with chemical methods for stabilizing the CsPbBr_3_ material. The growth method proposed in this work should be viable for a wide range of MHP materials. The demonstrated stability makes the vertically aligned CsPbBr_3_ NWs a promising foundation for a wide range of applications in many fields of optoelectronics.

## Supporting Information Available

Estimation of the maximum length of NWs in AAO pores, SEM-EDS spectra, further cross-sectional SEM, photos of samples, SEM of the sample surface, XRD details, further PL results, and XRD of stored samples (PDF)
