# Supplementary material for: Vertically Aligned CsPbBr3 Nanowire Arrays with Template-Induced Crystal Phase Transition and Stability
Source: J Phys Chem C Nanomater Interfaces. 2021 Feb 11;125(8):4860–8. doi: 10.1021/acs.jpcc.0c11217 (PMC7976601; doi:10.1021/acs.jpcc.0c11217)
Supplement: Supplementary file 1 — jp0c11217_si_001.pdf [file jp0c11217_si_001.pdf]

Electronic Supporting Information

Vertically Aligned $CsPbBr_3$ Nanowire Arrays with Template-induced Crystal Phase Transition and Stability

*Zhaojun Zhang[1], Klara Suchan[2], Jun Li[2], Crispin Hetherington[3], Alexander Kiligaridis[2], Eva Unger[2], Ivan G. Scheblykin[2], Jesper Wallentin[1]**

[1] Synchrotron Radiation Research and NanoLund, Department of Physics, Lund University, Box 124, Lund, 22100, Sweden.

[2] Chemical Physics and NanoLund, Department of Chemistry, Lund University, Box 124, Lund, 22100, Sweden.

[3] Centre for Analysis and Synthesis and NanoLund, Department of Chemistry, Lund University, Box 124, Lund, 22100, Sweden.

Corresponding author: jesper.wallentin@sljus.lu.se

S1. Estimation of the maximum length of NWs in AAO pores

The concentration of precursor is *c*, the molar weight of $CsPbBr_3$ *M*=579.82g/mol. The diameter and length of AAO pore is *d*=250 nm and l_0=5 μm, and we assume that the pore is ideally cylindrical shape. The density of $CsPbBr_3$ is ρ=4.75g/cm^3; [i]

We assume that the pore is completely filled with precursor, this means the precursor volume equals to the AAO pore volume. We also assume that all the precursor is used for one single nanowire growth. Then the mass of the $CsPbBr_3$ nanowire equal to the mass of $CsPbBr_3$ dissolved in the precursor.

Then the length of the $CsPbBr_3$ NW, *l,* will satisfy the following equation:

$$\rho \cdot \frac{\pi}{4} d^2 \cdot l = \frac{\pi}{4} d^2 \cdot l_0 \cdot c \cdot M$$

Then we can obtain that the ratio of NWs length to pore length is

$$l/l_0 = (c \cdot M)/\rho$$

Note that this ratio is independent of the diameter.

When the precursor concentration is *c*=0.5 M, we get $l/l_0 \approx 0.06$, which means that the NWs can only occupy 6% of the AAO pore.

If the pore length is 5 μm, then the theoretical length NWs is estimated to be about 300 nm. This value is close to the value we have obtained from the cross-sectional SEM images shown in Figure 1b.

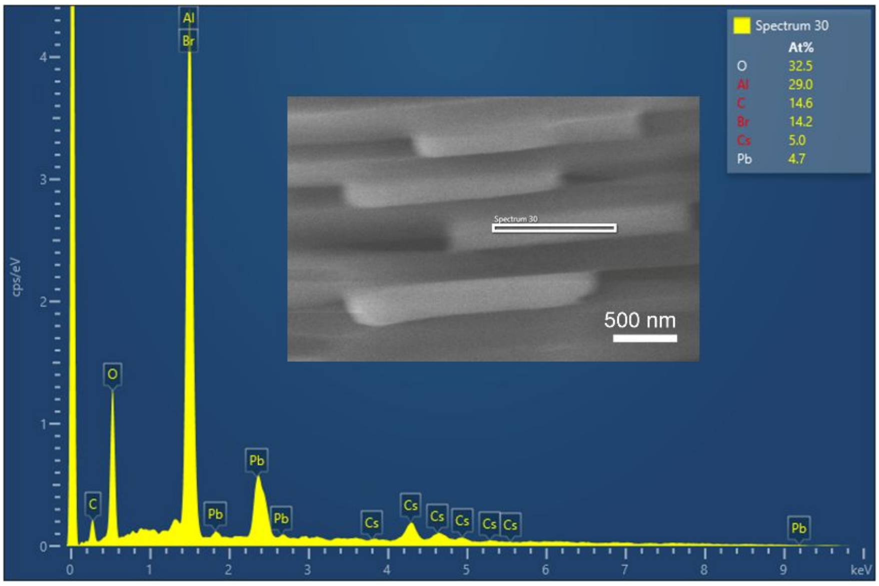

Figure S1. SEM-EDS spectra of $CsPbBr_3$ NW in AAO with diameter of 250 nm. The element atomic ratio of Cs:Pb:Br is about 5.0:4.7:14.2=1.06:1:3.02, which agrees well with the element ratio of $CsPbBr_3$. This confirms that the as-grown $CsPbBr_3$ NWs are $CsPbBr_3$.

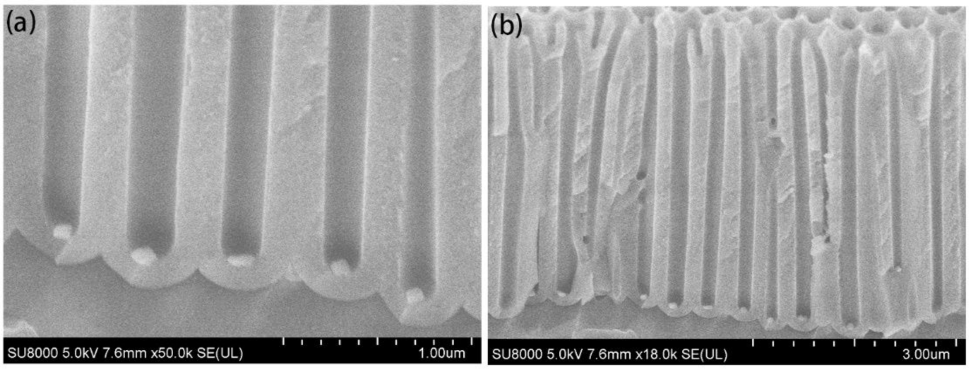

Figure S2: Small $CsPbBr_3$ nanoparticles with a size of around 100 nm in AAO with diameter of 250 nm which is grown by one-time spin coating 0.008 M precursor.

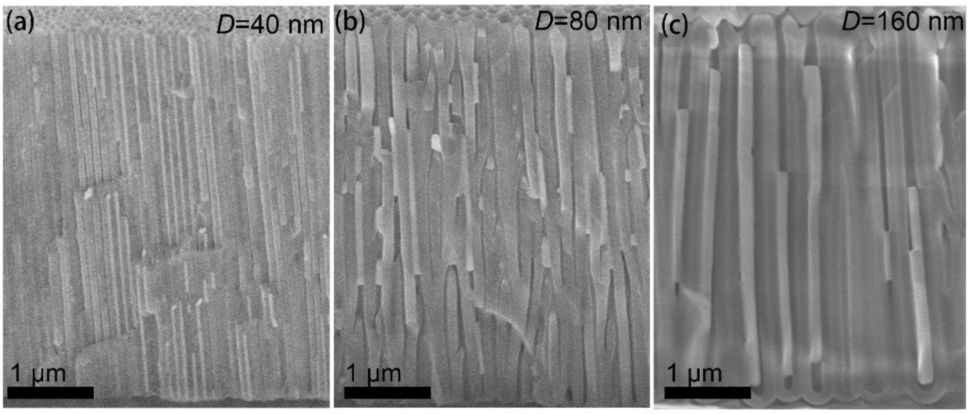

Figure S3: Cross-sectional SEM images of micrometer long $CsPbBr_3$-NWs/AAO with diameter D of 40, 80 nm 160 nm. For all the AAO templates, the pore length is 5 μm.

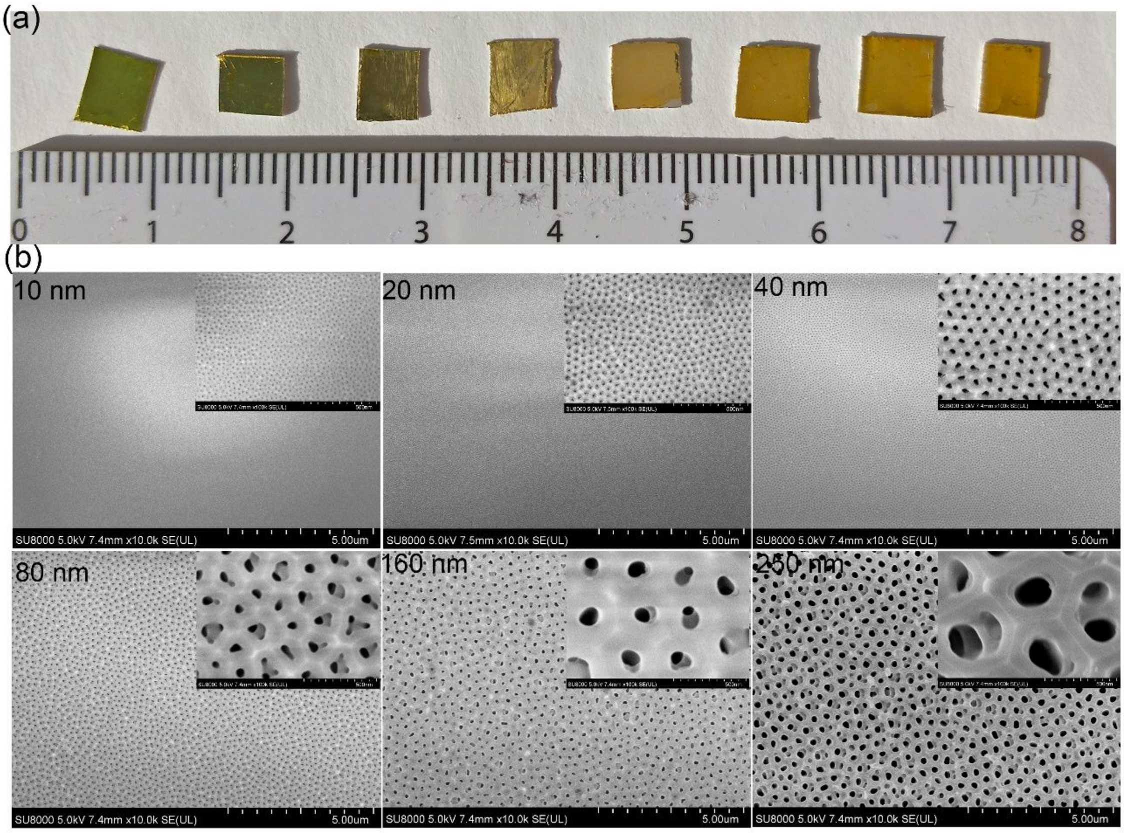

Figure S4 (a) Photos of the samples of $CsPbBr_3$-NWs/AAO with different diameter. **(b)** SEM images of **s**urface of $CsPbBr_3$-NWs/AAO samples with different diameters. Since these NWs were all grown close to the bottom part, the pores seem "empty" from the top view.

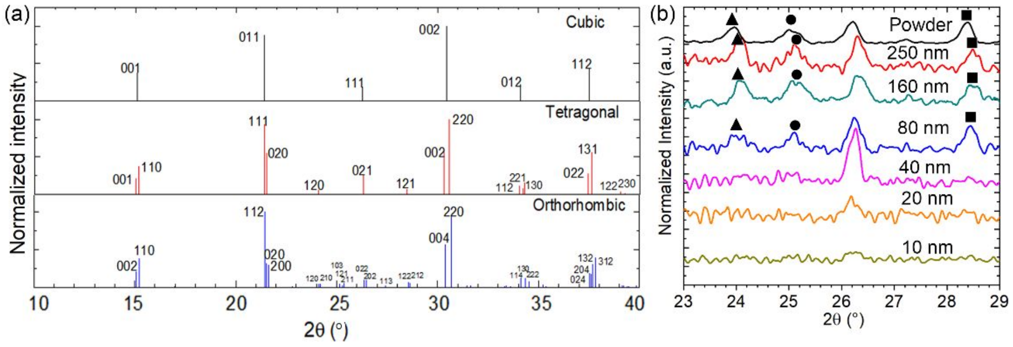

Figure S5. (a) Calculated powder XRD patterns of bulk orthorhombic (*Pnmb*, a = 8.207 Å, b = 8.255 Å and c = 11.759 Å), tetragonal (*P4mbm*, $a = b$ = 8.266 Å, c = 5.897 Å), and cubic (*Pm-3m*, $a = b = c$ = 5.871 Å) $CsPbBr_3$. (b) Measured XRD patterns of all the $CsPbBr_3$-NWs/AAO samples ranging from 23°-29°, which clearly show the structure transition of $CsPbBr_3$ NWs from orthorhombic to cubic phase.

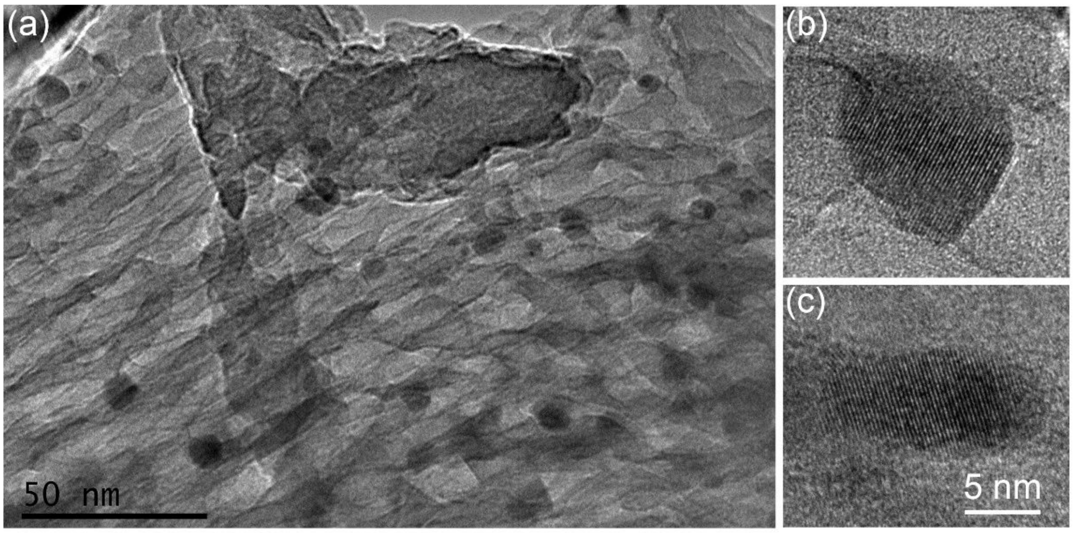

Figure S6. **(a)** Low magnification TEM image of $CsPbBr_3$-NWs/AAO with diameter of 10 nm. **(b)** and **(c)** High-resolution TEM images from regions of fig a. The lattice spacing is about 0.28 nm. It should be mentioned the NWs in pores become to "particle"-like shape due to beam damage.

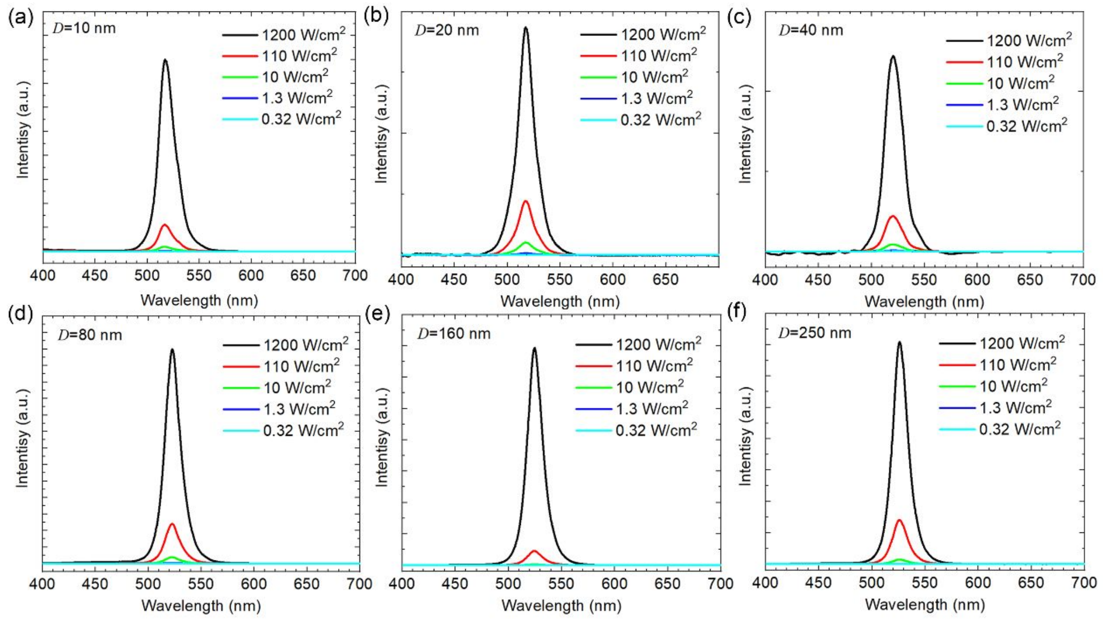

Figure S7 PL spectra of $CsPbBr_3$-NWs/AAO with different diameters *D* from 10 to 250 nm collected under excitation of increasing power density of 378 nm laser.

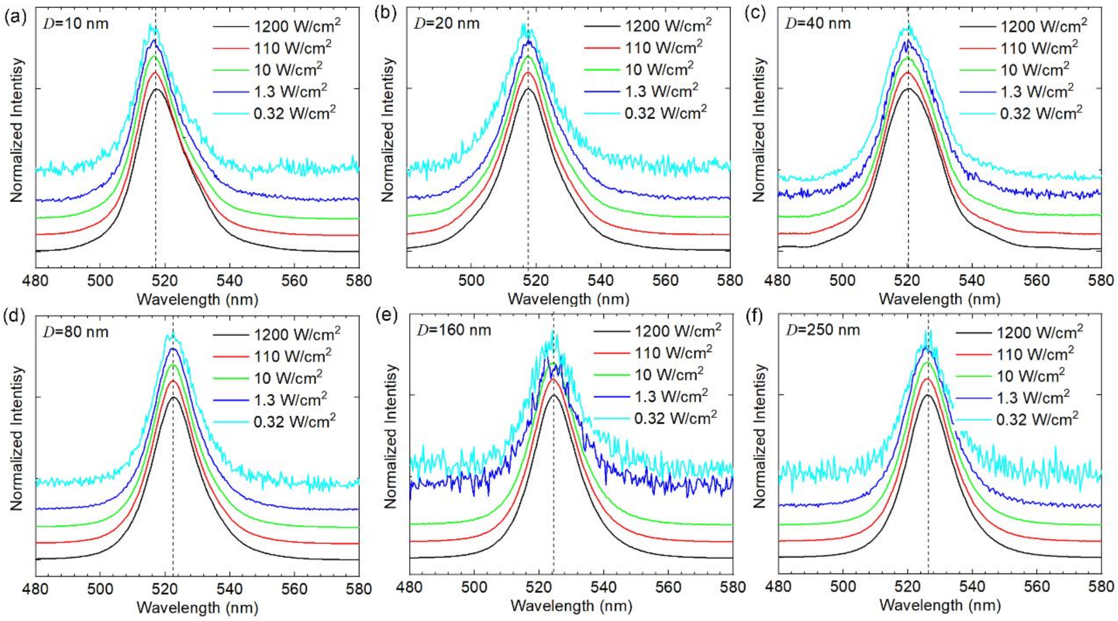

Figure S8 Normalized PL spectra of $CsPbBr_3$-NWs/AAO with different diameters collected under excitation of laser with increasing power density.

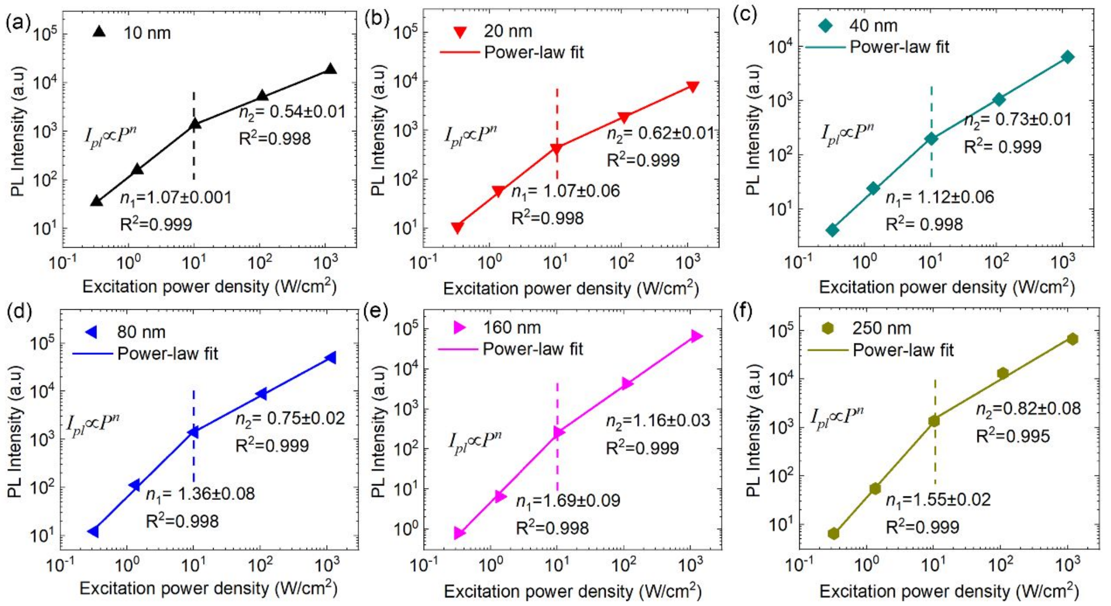

Figure S9: The fitting results of PL intensity versus excitation power density of $CsPbBr_3$-NWs/AAO with diameters from 10 to 250 nm.

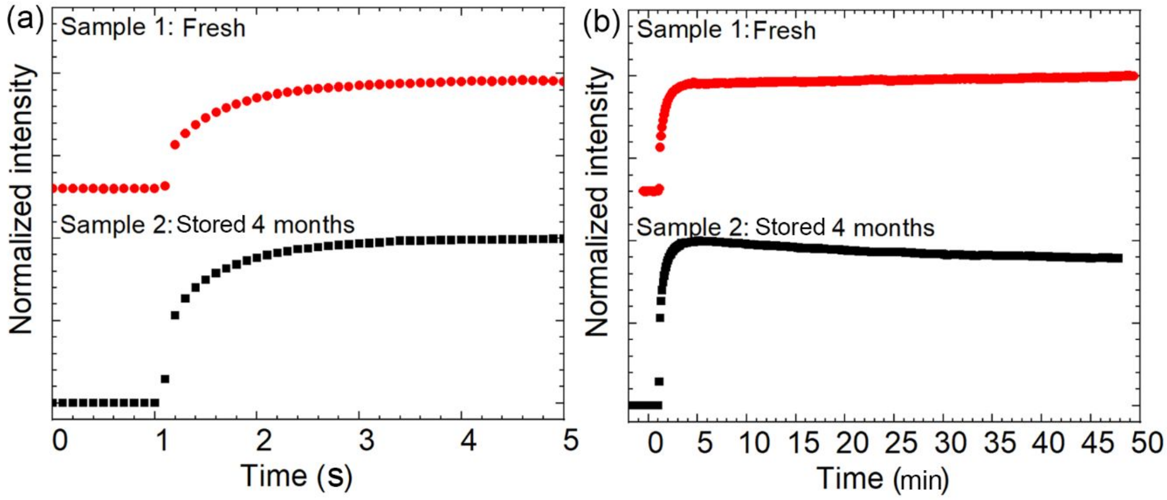

Figure S10 Time-dependent PL intensity test under 1 sun (0.1 W/cm^2) illumination by 485 nm laser for shorter time ranges than shown in the main text.

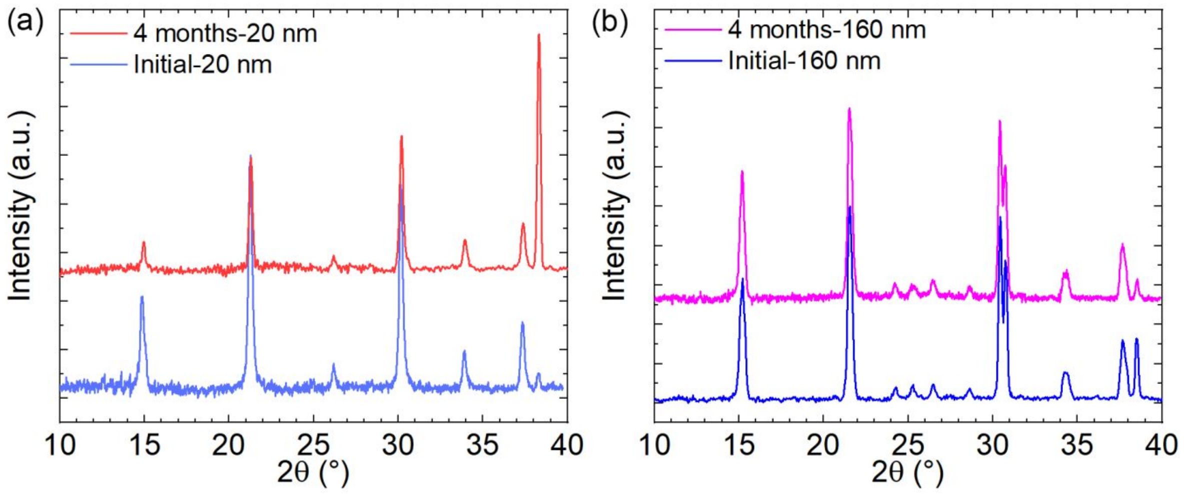

Figure S11 XRD patterns of $CsPbBr_3$-NWs/AAO with diameters of (a) 20 nm and (b) 160 nm after storage under ambient conditions (room temperature, humidity: 45%) for 4 months.

---

[i] $CsPbBr_3$ Crystal Structure: Datasheet from "PAULING FILE Multinaries Edition – 2012" in SpringerMaterials (https://materials.springer.com/isp/crystallographic/docs/sd_0545372), Springer-Verlag Berlin Heidelberg & Material Phases Data System (MPDS), Switzerland & National Institute for Materials Science (NIMS), Japan.
